# A Rare Case of Left Ventricular Non-Compaction with Coronary Artery Anomaly Complicated by ST-Elevation Myocardial Infarction and Subcutaneous Defibrillator Implantation

**DOI:** 10.3390/ijerph19020791

**Published:** 2022-01-11

**Authors:** Francesca Romana Prandi, Federica Illuminato, Chiara Galluccio, Marialucia Milite, Massimiliano Macrini, Alessio Di Landro, Gaetano Idone, Marcello Chiocchi, Francesco Paolo Sbordone, Domenico Sergi, Francesco Romeo, Francesco Barillà

**Affiliations:** 1 Unit of Cardiology, Department of Systems Medicine, University of Rome Tor Vergata, 00133 Rome, Italy; feilluminato@gmail.com (F.I.); chiaragalluccio91@gmail.com (C.G.); mari.milite@gmail.com (M.M.); massimilianomacrini@gmail.com (M.M.); aledil01@hotmail.com (A.D.L.); gaetano.idone@gmail.com (G.I.); domenicosergi@gmail.com (D.S.); romeocerabino@gmail.com (F.R.); francesco.barilla@uniroma2.it (F.B.); 2 Department of Diagnostic Imaging, Molecular Imaging, Interventional Radiology and Radiotherapy, University of Rome Tor Vergata, 00133 Rome, Italy; marcello.chiocchi@gmail.com (M.C.); sbordonefpaolo@gmail.com (F.P.S.); 3 Department of Departmental Faculty of Medicine, Unicamillus International Medical University of Rome, 00131 Rome, Italy

**Keywords:** left ventricular non-compaction, STEMI, coronary artery anomaly, heart failure, subcutaneous implantable converter defibrillator

## Abstract

Left ventricular non-compaction (LVNC) is a rare congenital cardiomyopathy caused by arrest of normal endomyocardial embryogenesis and characterized by the persistence of ventricular hypertrabeculation, isolated or associated to other congenital defects. A 33-year-old male, with family history of sudden cardiac death (SCD), presented to our ER with typical chest pain and was diagnosed with anterior STEMI. Coronary angiography showed an anomalous origin of the circumflex artery from the right coronary artery and a critical stenosis on the proximal left anterior descending artery, treated with primary percutaneous coronary intervention. The echocardiogram documented left ventricular severe dysfunction with lateral wall hypertrabeculation, strongly suggestive for non-compaction, confirmed by cardiac MRI. At 3 months follow up, for the persistence of the severely depressed EF (30%) and the family history for SCD, the patient underwent subcutaneous ICD (sICD) implantation for primary prevention. To the best of our knowledge, this is the first case of LVNC associated with anomalous coronary artery origin and STEMI reported in the literature. Arrhythmias are common in LVNC due to endocardial hypoperfusion and fibrosis. sICD overcomes the risks of transvenous ICD, and it is a valuable option when there is no need for pacing therapy for bradycardia, cardiac resynchronization therapy and anti-tachycardia pacing.

## 1. Introduction

Left ventricular non-compaction (LVNC) is a rare congenital cardiomyopathy thought to be caused by arrest of normal endomyocardial embryogenesis, leading to the persistence of numerous and deep trabeculations communicating with the ventricular cavity. This abnormality may be isolated (isolated ventricular non-compaction) or associated with other congenital defects, such as neuromuscular disorders, other cardiac anomalies and abnormal origin of coronary arteries [1,2]. The right ventricle may be involved (biventricular non-compaction). Compaction of the spongy network of fibers and intertrabecular recesses (or “sinusoids”) occurs between week 5 and 8 of embryonic development and proceeds from the base of the heart to the apex and from the epicardium to the endocardium; concurrently, coronary circulation develops, and the sinusoids are reduced to capillaries [1].

Both familial and sporadic forms of LVNC have been described. Genetic inheritance arises in at least 30–50% of patients [3]. Inheritance of LVNC is most often autosomal dominant or X-linked recessive, although autosomal recessive and mitochondrial (maternal) inheritance also occur. Most cases of LVNC associated with congenital heart defects present autosomal dominant inheritance in familial cases [4]. Different genes that cause LVNC have been identified, mostly sarcomeric and cytoskeleton encoding genes. In the case of LVNC associated with congenital heart disease, NOTCH signaling pathway seems to be affected. Mitochondrial genome mutations and chromosomal abnormalities have also been identified [3].

Clinical manifestations are variable, ranging from no symptoms to congestive heart failure (HF), arrhythmias, sudden cardiac death (SCD) and systemic thromboembolic events. Forty percent of patients present episodes of chest pain, usually in the absence of significant coronary artery lesions [5]. From the analysis of the coronary angiographies of 74 patients with LVNC, Stollberger and Finsterer detected coronary anomalies in seven patients and significant coronary lesions in only two patients [2]. Subendocardial perfusion defects at cardiac magnetic resonance imaging (MRI) and perfusion emission tomography (PET) scan and subendocardial ischemic lesions at postmortem analysis have been described in patients with LVNC [1]. Some authors have highlighted that a reduced coronary flow reserve (CFR, ratio of near maximal to basal myocardial flow) is often documented in LVNC patients. CFR reduction is not confined to the trabeculated segments, also extending to many nontrabeculated segments. At echocardiographic evaluation, CFR reduction is associated with segments with wall motion abnormalities. Epicardial stenosis is unlikely to explain the reduced CFR in LVNC patients, suggesting that a microcirculatory dysfunction may be responsible for contractile dysfunction and heart failure [1,6]. Therefore, in patients with LVNC, angina can have a dual origin since it may be secondary to a reduced CFR due to either atherosclerotic disease or microcirculatory dysfunction [7].

Diagnosis is frequently performed in early life, but it can also occur later in adulthood, and it is often underestimated. LVNC prevalence in adults is estimated at 0.014%, but a recent study showed that 25% of patients with LV dysfunction met the criteria for this diagnosis [8]. Diagnosis of LVNC can be made by color Doppler echocardiography, and it may be confirmed by cardiac MRI. Currently, the most widely used echocardiographic criteria diagnostic for LVNC are Jenni’s criteria, which include the coexistence of two separate layers, a thin compacted (C) epicardial layer and a thicker non-compacted (NC) endocardial layer with a NC/C ratio > 2 in end systole (parasternal short axis view), associated with the presence of color Doppler signal within the intertrabecular recesses [9]. The segments most frequently involved are the mid-lateral, mid-inferior and apical segments. These segments are often hypokinetic due to subendocardial hypoperfusion, reduced reserve of coronary flow and microcirculatory dysfunction [6,9]. Petersen et al. defined, as diagnostic criteria at cardiac MRI for pathological LVNC, a NC/C ratio > 2.3 in end diastole (long-axial sections) [10]. The diagnosis can be supported by clinical criteria, such as a first-degree family history of LVNC, the association with neuromuscular disease and other cardiac malformations, a past medical history of thromboembolic events, the presence of regional kinetic anomalies and malignant arrhythmias. It is recommended to screen for familial LVNC in first-degree relatives of index cases.

## 2. Case Report

**Figure 1:**
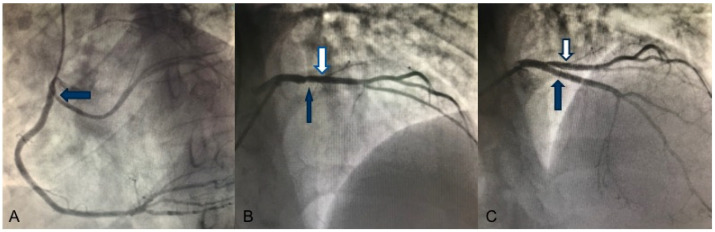
Coronary angiography. (**A**) Left oblique angiographic view: anomalous origin of the LCx (blue arrow) from the RCA. (**B**) Anteroposterior cranial angiographic view: large-caliber diagonal branch (white arrow) and critical stenosis of the proximal LAD artery that appears not viewable (blue arrow) (**C**) Anteroposterior cranial angiographic view: result after primary PCI and a DES implantation in proximal LAD (blue arrow) with effective restoration of the vessel’s patency and downstream flow. Large-caliber diagonal branch (white arrow).

A 33-year-old male, smoker, with family history for sudden cardiac death (father deceased at 54 years old) presented to the emergency room for typical chest pain radiated to the left arm, unrelated to exertion. The physical examination was within normal limits. The laboratory exams showed increased cardiac enzymes levels (Troponin I hs 49,308.7 ng/L, Myoglobin 2494 ng/mL, Creatine kinase-MB 252 ng/mL). The electrocardiogram (EKG) showed ST elevation in the anterior leads and diffuse ventricular repolarization anomalies, suggestive of anterior ST elevation myocardial infarction (STEMI). The patient underwent coronary catheterization, with evidence of anomalous origin of the left circumflex artery (LCx) from the Right Coronary Artery (RCA) (Figure 1A) and critical stenosis on the proximal left anterior descending artery (LAD) (Figure 1B), treated with primary percutaneous coronary intervention (PCI) and implantation of a drug-eluting stent (DES) with a good angiographic result (Figure 1C). 

**Figure 2:**
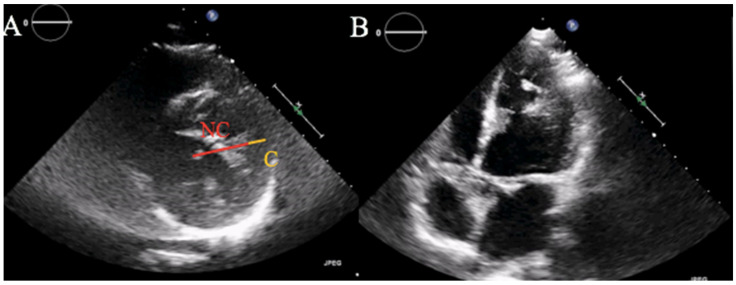
Transthoracic echocardiogram. (**A**) Parasternal short axis view showing an end systolic NC/C ratio > 2, diagnostic for LVNC according to Jenni’s criteria. (**B**) Apical four chambers view with evidence of hypertrabecular appearance of the left ventricle at the apex and lateral wall.

The patient was transferred to the Cardiac Intensive Care Unit. Blood tests showed low-density lipoprotein (LDL) cholesterol of 137 mg/dL and normal glycosylated hemoglobin. Transthoracic echocardiogram (TTE) documented moderate left ventricular (LV) dilatation, severe LV dysfunction (ejection fraction 30%) with apical, septal and anterior wall akinesis, and lateral wall hypertrabecularization with multiple prominent trabeculations and deep intertrabecular recesses communicating with the ventricular cavity, with an end systolic NC/C ratio > 2 in parasternal short axis view, suggestive for LVNC (Figure 2A,B). 

**Figure 3:**
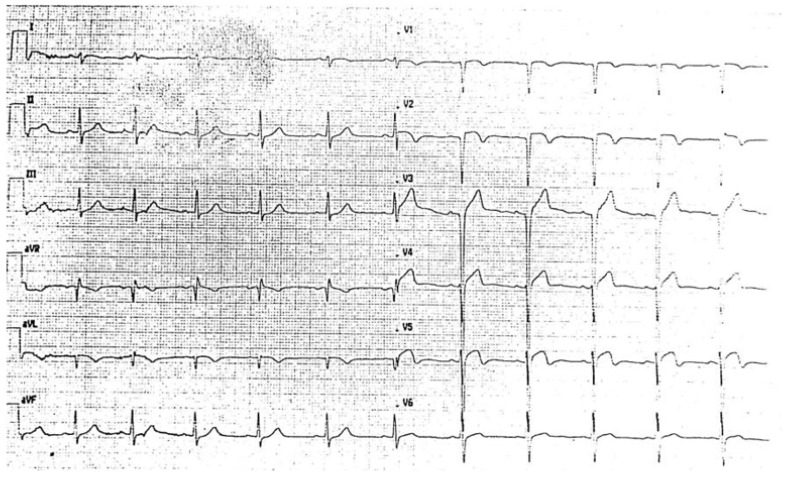
EKG on 3rd day after reperfusion documented QS complexes in the anterior leads and diffuse ventricular repolarization abnormalities, with preserved atrio-ventricular and intra-ventricular conduction.

EKG on the 3rd day after reperfusion showed QS complexes in the anterior leads and diffuse ventricular repolarization abnormalities, with preserved atrio-ventricular and intra-ventricular conduction (Figure 3). 

**Figure 4:**
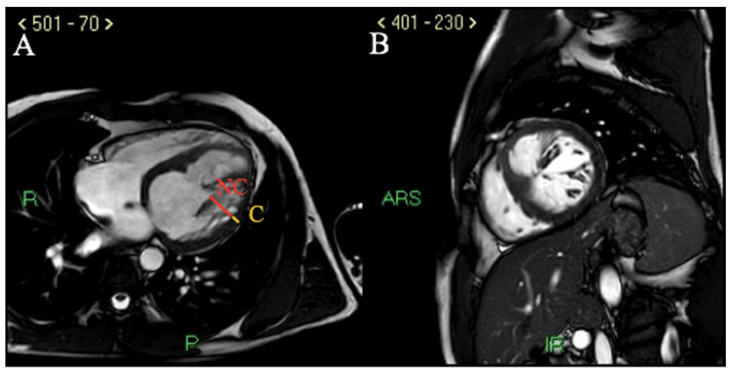
Cardiac MRI. (**A**) cine-steady state-free precession sequence (SSFP) showing an end diastolic NC/C ratio of 5 in long axis view, diagnostic for pathologic LVNC according to Petersen’s criteria. (**B**) cine-SSFP sequence showing non-compaction myocardial hypertrabecularization in end diastolic short axis view.

Cardiac MRI documented dilated LV with ejection fraction (EF) 34%, anterior and antero-septal wall akinesis (associated with increased T1 mapping values and areas of late gadolinium enhancement or LGE after contrast injection, compatible with ischemic pattern), infero-lateral wall hypokinesia and LV free wall marked hypertrabecularization with a telediastolic NC/C ratio of 5 in long-axis view (Figure 4A,B).

The right ventricle (RV) was normal in size and function, and there were no significant valvular alterations. The patient was given dual antiplatelet therapy (Ticagrelor 90 mg b.i.d. and Cardioaspirin 100 mg per day), beta-blocker (Bisoprolol 5 mg o.d.), ACE-inhibitor (Ramipril 5 mg per day), loop diuretic (Furosemide 25 mg b.i.d.), mineralocorticoid receptor antagonist (Spironolactone 25 mg o.d.) and statin medications (Atorvastatin 80 mg o.d.). During hospitalization, ACE-inhibitor was switched to angiotensin receptor-neprilysin inhibitor (ARNI) with Sacubitril/Valsartan (24/26 mg b.i.d.) after a washout period of 36 h. After a week, Sacubitril/Valsartan was titrated to 49/51 mg b.i.d., and Furosemide was reduced to 25 mg o.d. A prolonged EKG monitoring with telemetry was performed since admission, which did not document any significant arrhythmias. The patient was discharged in stable clinical conditions. At two months cardiologic follow-up visit, the patient was asymptomatic for chest pain, but presented symptomatic heart failure (NYHA class II) and the TTE confirmed a dilated LV with severely depressed EF (30%). ARNI therapy was titrated to target dose (Sacubitril/Valsartan 97/103 mg/die), Furosemide was reduced to 25 mg o.d. only 4 days per week and Spironolactone was titrated to 50 mg o.d. since serum potassium level was 4.6 mEq/L. Sodium–glucose co-transporter 2 inhibitors were not introduced into therapy, since after ARNI titration to target dose, the patient systolic blood pressure was between 100 and 110 mmHg and NT-pro BNP level was within normal range. In consideration of the post-ischemic dilated cardiomyopathy with severely depressed EF and family history of SCD (his father deceased at 54 years old), an implantable cardioverter defibrillator (ICD) in primary prevention was recommended. The presence of a narrow QRS (<120 ms), a preserved atrio-ventricular conduction and the absence of any bradyarrhythmia nor any monomorphic ventricular tachycardia previously reported supported the decision for implantation of a subcutaneous implantable cardioverter defibrillator (sICD) that was performed at 3 months from previous discharge without any periprocedural complications. The patient was discharged in stable conditions with a remote home monitoring transmitter.

## 3. Discussion

### 3.1. LVNC and Coronary Artery Anomalies

The incidence of coronary artery anomalies in LVNC patients is unknown due to the rarity of both conditions. Major epicardial coronary arteries distribution and size are strictly related to their dependent myocardium extent. During cardiac development, lack of coronary blood flow would produce hypoplasia of the dependent myocardial mass, and conversely a reduction in the myocardial mass would induce hypoplasia of the relative coronary branch. Coronary ostia are formed soon after truncal separation, while the distal coronary arteries remain as a loose network until myocardial mass develops during later developmental stages [11]. Left ventricular trabecular compaction occurs between week 5 and 8 of embryonic development concurrently with the invasion of the myocardium by the developing coronary vasculature coming from the epicardium, and the intertrabecular recesses are transformed in capillaries [1]. Planar cell polarity gene (PCP), named Vangl2, and RhoA/Rho kinase signaling may be important at different levels for development of the mature ventricular myocardium and the coronary vessels. Vangl2 is expressed exclusively in the myocardiocytes of the developing heart, and it is not found in the coronary progenitors. Loss of functional Vangl2 results in reduced fibronectin deposition in the subepicardial space, with limited migration of epicardially derived cells into the myocardium and disruption of RhoA/Rho kinase signaling with cytoskeletal disorganization. Mice fetuses with Vangl2 disruption display abnormal myocardial polarity, cytoskeletal disorganization, and altered ventricular thickening. Moreover, they also present abnormal coronary artery development with abnormal smooth muscle cell layer and enlarged, ectopic vessels. Inhibition of myocardial Rho kinase produces the same spectrum of defects, affecting both cardiomyocytes organization and coronary vasculature formation [12].

Rare cases of anomalous coronary artery origin in patients with LVNC have been reported in the literature. Mattson et al. reported a case of a patient with LVNC and anomalous origins of the LCx from the RCA and reviewed other cases of anomalies in LVNC patients previously described in the literature, including a single coronary artery of anomalous origin and four arteries arising from the RCA with an LCx arising directly from the aorta [13]. The anomaly found in our patient (LCx origin from RCA) represents the second most common coronary artery anomaly in the general population (the most common is a separate origin of LAD and LCx), and it is reported in 0.37% of coronary angiographies. It is generally not linked to an increased risk of SCD, except for when the LCx travels between the aorta and the pulmonary artery, where LCx angulation or lateral compression may cause increased SCD risk [11].

Further studies are needed in order to assess a possible common pathophysiological background between LVNC and coronary artery abnormalities, but until now, we should consider our case as only an association between two independent rare conditions.

### 3.2. LVNC and Acute Coronary Syndrome

LVNC is sometimes associated with coronary artery disease (CAD), but only rare cases of acute coronary syndrome (ACS) have been described in the literature, with exceptionally rare cases of LVNC incidental diagnosis after STEMI, such as in our patient. To the best of our knowledge, this is the first case of LVNC associated with anomalous coronary artery origin and STEMI reported in the literature. Seven cases of acute myocardial infarction (AMI) have been reported in patients with LVNC [14,15,16,17,18,19,20], five of them were anterior STEMI in male patients aged between 45 and 63 years [14,16,17,18,19], and one was an AMI in a patient with three-vessels disease treated with coronary artery bypass graft (CABG) [20]. Panduranga et al. reported a case of a patient with LVNC and STEMI, non-dyslipidemic, and hypothesized that a single gene responsible for both myocardial development and coronary endothelium could be involved in the pathogenesis of LVNC and at the same time predisposition to coronary atherosclerosis [14]. Swinkels et al. described a case of subacute AMI in a LVNC patient treated with CABG [21]. Fettouhi et al. reported a case of a patient that presented with convulsive crises and was diagnosed with LVNC and apical AMI [22]. A case of cardiac arrest and severe three-vessels disease associated with LVNC and severe mitral regurgitation treated with CABG and mitral valvuloplasty was described by Salati et al. [23]. Martini et al. reported a case series of familial LVNC associated with familial dyslipidemia and family history of documented CAD, discussing that the association between LVNC and CAD may be linked to familial dyslipidemia [24]. Finally, Finsterer et al. reported the case of an old male with LVNC, metabolic myopathy and CAD diagnosed at autoptic exam [25].

Further studies are necessary in order to assess the possible pathophysiological mechanisms that correlate LVNC and coronary atherosclerosis.

### 3.3. LVNC and Arrhythmic Risk

Arrhythmias and conduction system disease are common in patients with LVNC. The mismatch between myocardial mass and number of capillaries in LVNC determines endocardial myocardium hypoperfusion, despite normal epicardial coronary arteries and asynchronous contraction between compacted and non-compacted layers that lead to systolic dysfunction. Moreover, trabeculations reduce ventricular compliance, leading to diastolic dysfunction. Ischemia is responsible for the development of progressive fibrosis, which contributes to the decrease in the systolic function and predisposition to ventricular arrhythmias [1].

A wide spectrum of cardiac arrhythmias has been observed in patients with LVNC, including atrioventricular conduction diseases and supraventricular and ventricular tachyarrhythmias. Atrial fibrillation is reported in more than 25% of LVNC adult patients [1] and ventricular tachyarrhythmias in 38–47% of LVNC adults and in 13–18% of those who die suddenly [3]. The presence of arrhythmias is an independent risk factor for mortality.

Ventricular arrhythmias described in this patient population include monomorphic ventricular tachycardia (VT), bidirectional VT, polymorphic VT and ventricular fibrillation (VF). Therefore, patients with LVNC may present higher risk for SCD [26]. Electrophysiologic (EP) study can be used to risk stratify patients and guide therapy (medication versus ICD implantation) in patients with inducible VTs. Steffel et al. investigated the prognostic significance of findings during EP study in 24 patients with LVNC and documented inducible ventricular arrhythmias in nine patients (38%), of which seven underwent ICD implantation, with three of them experiencing ventricular arrhythmias treated with anti-tachycardia pacing (ATP) or shock over a follow-up of 61 months. [27]. Further studies including a long-term follow-up are necessary to assess the role of EP testing for arrhythmic risk stratification in these patients. EP study or ICD implantation should be considered for these patients, especially if they present any ventricular arrhythmias or a reduced LVEF [26].

According to the recommendations for device therapy in HF with reduced EF (EF ≤ 40%) in current European Society of Cardiology (ESC) guidelines, an ICD for primary prevention is indicated (class I indication) in case of ischemic etiology and should be considered (class IIa indication) in cases of non-ischemic etiology in order to reduce the risk of SCD and all-cause mortality in patients with symptomatic HF (NYHA class II-III) and an LVEF ≤ 35% despite ≥ 3 months of optimal medical therapy (OMT), provided they are expected to survive substantially longer than 1 year with good functional status [28]. Risk stratification of SCD is one of the major unresolved issues of modern cardiology. Current guidelines identify ejection fraction and NYHA class as the only instrumental parameters for risk stratification of sudden cardiac death. It is strongly suggested from clinical trials that ejection fraction reduction is the key predictor of total mortality and sudden death regardless of its etiology; however, it cannot be considered as an indisputable gold standard predictor of risk because it lacks sensitivity and specificity in the prediction of sudden death. It is reasonable that many factors besides EF influence patient prognosis; there are different aspects suggesting that a reduction in EF is a risk factor only in combination with other risk and prognostic factors [29].

According to the ESC guidelines on ventricular arrhythmias and SCD prevention, LVNC in the absence of additional risk factors is not an indication for the implantation of the ICD for primary prevention, and for the assessment of ICD need, it is recommended to follow the criteria used for dilated cardiomyopathy [30]. ICD implantation is indicated in patients with LVNC and cardiomyopathy with severe LV dysfunction (EF < 35%), a previous history of sustained ventricular tachycardia or fibrillation, recurrent syncope of unknown origin, or a family history of SCD [3]. Increased age, increased LV end diastolic diameter, atrial fibrillation, bundle branch block, symptomatic HF and associated neuromuscular disease are reported predictors for increased mortality [30].

Involvement of the RV in LVNC cannot be excluded, even when it appears normal on cardiac MRI, since the distinction from normal RV trabeculations is difficult [31]. In cases of LVNC, especially in cases of RV involvement, there might be higher risk of cardiac perforation by the ICD lead. sICD may potentially overcome the risks of transvenous ICD (such as endocarditis, tricuspid regurgitation and ventricular perforation) since the extrathoracic placement of the sICD makes it impossible for the device to deliver pacing therapy. sICD should be considered (class indication II a) as an alternative to transvenous defibrillators in patients with an indication for an ICD when pacing therapy for bradycardia support, cardiac resynchronization or ATP is not needed [30].

Until now, sICD implantation has been reported in the literature in only 14 patients with LVNC, mainly in children and young adults, and only two received inappropriate shock deliveries, due to atrial fibrillation with high ventricular response, and were therefore switched to transvenous ICD to have better discrimination of the arrhythmia origin [27]. In children, antiarrhythmic drugs may be indicated before ICD because of the high risk of lead fracture and inappropriate shocks in this population [3].

In our patient, EP study was not performed since the indication for ICD in primary prevention was already driven by the post-ischemic dilated cardiomyopathy with severely depressed EF (30%) with symptomatic heart failure (NYHA class II) despite optimal medical therapy at two months follow up after the STEMI, in consideration also of the patient’s family history for SCD. The presence of a narrow QRS (<120 ms), a preserved atrio-ventricular conduction, and the absence of any previously reported bradyarrhythmia nor monomorphic ventricular tachycardia episodes, supported the decision for implantation of a sICD in primary prevention.

### 3.4. LVNC Diagnostic and Prognostic Challenges

A systematic review and meta-analysis of outcomes and prognoses associated with LVNC were performed by Aung et al. They observed a pooled event rate for cardiovascular mortality of 1.92 per 100 person-years, similar to the control group with dilated cardiomyopathy (odds ratio, 1.10) and 25-fold higher than the general population [32]. The risks of all cause-mortality, thromboembolism and ventricular arrhythmias were found to be similar to those with dilated cardiomyopathy, although patients with LVNC had a higher incidence of heart failure hospitalization. Low LVEF, and not the extent of trabeculation, appeared to be associated with worse outcomes. However, there was substantial heterogeneity between the studies analyzed in this meta-analysis [32] that may be due to differences in LVNC diagnostic criteria and modality used for the diagnosis, in consideration of the lack of unified diagnostic criteria [33]. Grigoratos et al. performed a meta-analysis evaluating, by cardiac MRI, the prognostic role of LGE and global systolic impairment in patients with LVNC. They reported that when matched for LVEF, patients with and those without LVNC have similar prognoses, while LGE is associated with worse prognosis in patients with LVNC independent from LVEF [34]. In patients with LVNC, LVEF > 50% and negative LGE with no cardiac death, sudden cardiac death, resuscitated cardiac arrest and appropriate ICD firing occurred, highlighting the importance of cardiac MRI not only for the diagnosis but also for risk stratification and future therapeutic management [34,35,36,37]. The heterogeneity in characterizing LVNC represents a challenge to define reliable diagnostic criteria. The presence of LV hypertrabeculation is not necessarily evidence of cardiomyopathy. Prevalence of LVNC was significantly higher in cohorts defined by cardiac MRI (14.79%) compared with echocardiography (1.28%), but this could also lead to potential overdiagnosis [38]. LV systolic impairment and LGE inclusion among the diagnostic criteria for true LVNC cardiomyopathy need to be considered for a more standardized assessment of LVNC (diagnosis of true LVNC vs. hypertrabeculation), with consequent better prognostic definition [34]. An expert consensus is needed to harmonize the diagnostic criteria, risk factors and prognostic factors in LVNC in order to develop a more standardized assessment [32].

## 4. Conclusions

We reported a rare case of LVNC and coronary artery anomaly incidental diagnosis after STEMI, complicated by heart failure and subsequent implantation of sICD in primary prevention.

LVNC can be isolated or associated with other extra-cardiac and cardiac abnormalities, including coronary arteries anomalies. Many patients with LVNC are asymptomatic, but some present with HF, thromboembolism, arrhythmias or SCD, coronary artery disease and rarely acute coronary syndromes. Further studies are necessary in order to assess the possible pathophysiological mechanisms that correlate LVNC and coronary atherosclerosis. Echocardiography, integrated in doubtful cases with multimodality imaging assessment (especially with cardiac MRI), represents the main diagnostic tool in LVNC, and it is also important for screening of familial LVNC in first-degree relatives of index cases.

To the best of our knowledge, this is the first case of LVNC associated with anomalous coronary artery origin and STEMI reported in the literature. We discussed the association of LVNC with coronary artery anomaly, coronary atherosclerosis, ACS and with arrhythmias, reporting the current guidelines for ICD implantation in LVNC patients. sICD overcomes the risks of transvenous ICD, and it is a valuable option in adult patients with LVNC not requiring pacing for bradycardia, cardiac resynchronization therapy or anti-tachycardia pacing.

## Data Availability

No new data were created or analyzed in this study. Data sharing is not applicable to this article.

## Abbreviations

LVNC = left ventricular non-compaction; HF = heart failure; SCD = sudden cardiac death; MRI = magnetic resonance imaging; PET = perfusion emission tomography; CFR = coronary flow reserve; C = compacted; NC = non-compacted; EKG = electrocardiogram; STEMI = ST elevation myocardial infarction; LCx = left circumflex artery; RCA = right coronary artery; LAD = left anterior descending artery; PCI = percutaneous coronary intervention; DES = drug-eluting stent; LDL = low-density lipoprotein; TTE = transthoracic echocardiogram; LV = left ventricular; EF = ejection fraction; LGE = late gadolinium enhancement; RV = right ventricle; ACE-inhibitor = angiotensin converting enzyme-inhibitor; ARNI = angiotensin receptor-neprilysin inhibitor; NYHA = New York Heart Association; NT-pro BNP = N-terminal-proB-type Natriuretic Peptide; ICD = implantable cardioverter defibrillator; sICD = subcutaneous implantable cardioverter defibrillator; PCP = planar cell polarity gene; CAD = coronary artery disease; ACS = acute coronary syndrome; AMI = acute myocardial infarction; CABG = coronary artery bypass graft; VT = ventricular tachycardia; VF = ventricular fibrillation; EP = electrophysiologic; ATP = antitachycardia pacing; LVEF = left ventricular ejection fraction; ESC = European Society of Cardiology; OMT = optimal medical therapy.
